# The Rarest of the Rare: Crossed Fused Renal Ectopia of the Superior Ectopia Type

**DOI:** 10.1155/2015/742419

**Published:** 2015-04-29

**Authors:** Leyla Akdogan, Ali Kemal Oguz, Tarkan Ergun, Ihsan Ergun

**Affiliations:** ^1^Department of Internal Medicine, Ufuk University School of Medicine, Ankara, Turkey; ^2^Dr. Rıdvan Ege Hospital, Konya Bulvarı No. 86-88, Balgat, Çankaya, 06520 Ankara, Turkey; ^3^Department of Radiology, Baskent University School of Medicine, Alanya, Antalya, Turkey; ^4^Baskent University School of Medicine, Alanya Research and Education Hospital, Alanya, 07400 Antalya, Turkey; ^5^Division of Nephrology, Department of Internal Medicine, Ufuk University School of Medicine, Ankara, Turkey

## Abstract

Crossed fused renal ectopia is a rare congenital anomaly of the urinary system where one kidney crosses over to opposite side and the parenchyma of the two kidneys fuse. Herein, we present an atypical CFRE case whose renal anatomy does not exactly match any of the already defined CFRE types. Both of the kidneys are ectopic with the crossed ectopic right kidney lying superiorly and fused to the upper pole of the left kidney. Renal arteries were originating from the common iliac arteries. A focal 90% stenosis was observed on the right main renal artery. The patient is borderline hypertensive.

## 1. Introduction

Crossed fused renal ectopia (CFRE) is a markedly rare congenital malformation of the urinary system where one of the kidneys crosses the midline to become located on the opposite side of its ureter entrance to the bladder and the parenchymas of the two kidneys fuse. CFRE has a reported autopsy incidence of around 1 : 2000 and is the second most frequently observed fusion anomaly of the kidneys following the horseshoe kidney. Resulting from aberrant migration and crossing of the midline of the metanephric blastema and the ureteral bud, CFRE is thought to develop during the fourth to eighth weeks of gestation. Mostly remaining asymptomatic and detected as an incidental finding during imaging studies, six well-defined anatomical variations of CFRE have been reported [1, 2]. Herein, we present an atypical CFRE case whose renal anatomy does not exactly match any of the already defined CFRE types.

## 2. Case Presentation

A 25-year-old man with no current complaints was referred to our nephrology outpatient clinic on the occasion of an incidental finding of renal ectopia. His initial abdominal ultrasonography had reported renal ectopia with both kidneys located in the left iliac fossa. Except a blood pressure reading of 135/85 mmHg, a complete physical examination was nonrevealing. His body mass index was within the normal range. Routine laboratory studies did not document any abnormalities (i.e., blood urea nitrogen: 9 mg/dL, creatinine: 0.95 mg/dL, normal serum electrolyte concentrations, normal urinalysis). A repeated renal ultrasound documented two ectopic kidneys located superiorly and inferiorly in the same anatomic location. Renal dimensions and parenchymal thicknesses of the superiorly and the inferiorly situated kidneys were 115 × 36 × 33/11 mm and 116 × 59 × 53/21 mm, respectively. The superiorly located kidney also displayed irregular contours and a thinner parenchyma, especially prominent in its superior pole. No renal calculi or dilatation of the collecting systems was observed.

**Figure 1:**
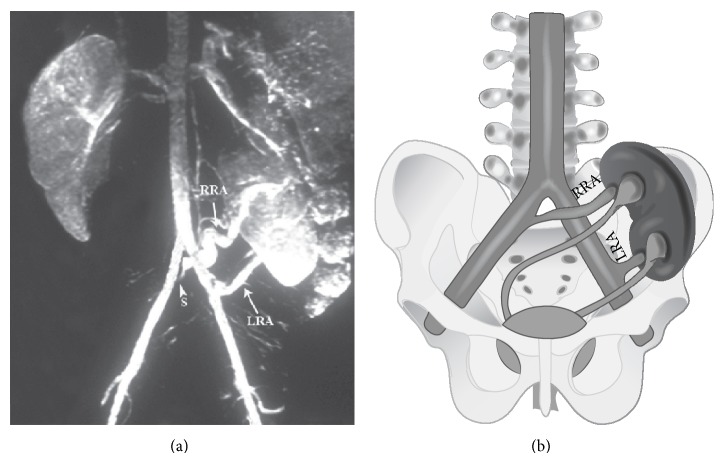
(a) The arterial phase of the 3D gadolinium-enhanced aortoiliac and renal magnetic resonance angiography of the patient. RRA and LRA (arrows) are pointing to the right and left main renal arteries, respectively, whereas S (arrow head) is marking the stenotic lesion closely located to the origin of the right main renal artery. (b) Schematic drawing of the renal and renal artery anatomy of the patient. Again, RRA and LRA are the right and left main renal arteries, respectively.

In order to exclude “white coat” hypertension, an ambulatory 24 hour blood pressure monitoring was performed which documented a mean blood pressure of 129/78 mmHg compatible with a borderline hypertension (the 24 hour average was higher than the expected limit of 115/75 mmHg, close to the upper limit of 130/80 mmHg). Accordingly, in search of a renovascular etiology of hypertension, renal Doppler ultrasonography was performed. Excluding elevated renal resistive index values in both renal arteries, the Doppler study was not informative. A 3D gadolinium enhanced aortoiliac and renal magnetic resonance angiography was planned and it revealed the exact renal and renal vascular anatomy of the patient. According to the findings of the angiography, the left ectopic kidney was located in the left iliac fossa with the crossed ectopic right kidney lying superiorly and fused to the upper pole of the left kidney. Also, both main renal arteries were originating from the corresponding ipsilateral common iliac arteries and there was a focal 90% stenosis situated 5 mm distal to the origin of the right main renal artery (Figure 1).

With the aim of documenting the potential contribution of the stenotic lesion to the borderline hypertension, plasma renin activity (24.5 pg/mL (normal values, 5.41–34.53 pg/mL)) and aldosterone level (149 pg/mL (normal values, 30–160 pg/mL)) were measured. Additionally, a captopril Tc99m-MAG3 radioisotope renography was performed, revealing a right: left differential renal function of 38 : 62 and no depression or alteration of renal functions following the test dose of captopril. Consequently, no immediate intervention for the stenotic lesion was planned, whereas a close followup, the DASH diet, and therapeutic lifestyle modification were strongly advised. Any plans of renal artery intervention were reserved for future use in cases of increasing blood pressure or the emergence of overt hypertension. The patient's blood pressure is still under control without the need of any drug therapy.

## 3. Discussion

**Figure 2:**
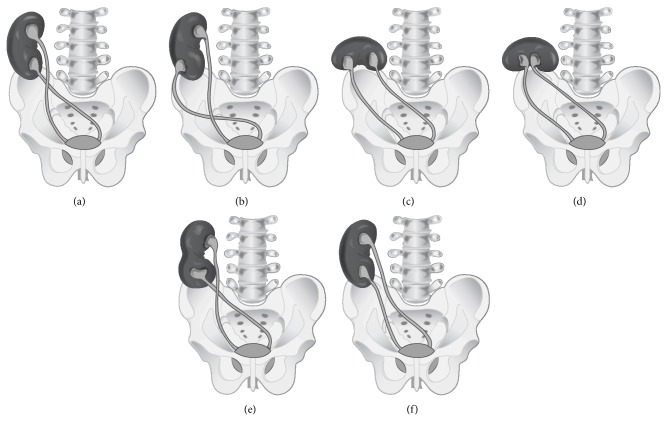
Six anatomical variations (types) of crossed fused renal ectopia: (a) inferior crossed fused renal ectopia; (b) sigmoid or S-shaped kidney; (c) lump kidney; (d) disc kidney; (e) L-shaped kidney; (f) superior crossed fused renal ectopia.

Crossed renal ectopia is classified into 4 main categories: crossed renal ectopia with or without fusion, unilateral crossed renal ectopia (with unilateral renal agenesis), and bilateral crossed renal ectopia (without fusion) [3]. In 85–90% of the crossed renal ectopia cases, the kidneys are partially or completely fused, hence given the name CFRE. CFRE is reported to be two times more prevalent in men than women [4]. Consistently, the patient we presented was also a young man. With respect to CFRE, six anatomical variations have been described [1], namely, inferior CFRE, sigmoid kidney, lump kidney, disc kidney, L-shaped kidney, and superior CFRE (Figure 2). While the inferior CFRE is the most frequent type observed, the superior CFRE is reported to be the least common. In the inferior CFRE type, the upper pole of the inferiorly situated crossed ectopic kidney is fused to the lower pole of the superiorly, normally positioned kidney. Another characteristic feature of CFRE is the three times more common occurrence of left-to-right ectopy [2]. Noteworthily, in our case both kidneys were ectopic and the crossed ectopic right kidney was positioned superiorly with its lower pole fused to the upper pole of the left kidney. To our best knowledge, in the current literature, there is only one case reported with superior CFRE of the right kidney [5].

As previously mentioned, most CFRE cases remain asymptomatic and are detected incidentally. This was also the case for our patient. When present, hydronephrosis, recurrent urinary tract infections, and renal calculi are the main reported complications of CFRE. Also, there may be associating congenital malformations affecting the urogenital, gastrointestinal, and musculoskeletal systems and vesicoureteral reflux is the most common accompanying urogenital abnormality [6, 7]. The only case with superior CFRE of the right kidney previously reported by Yin et al. had also associating retroiliac megaureter and thoracic scoliosis anomalies [5]. In our case, CFRE was present as an isolated congenital malformation.

Hypertension is very rare in CFRE and there is no given single pathophysiologic mechanism for this entity in the literature [8]. Our patient had a borderline hypertension and a stenosis was documented in the proximal segment of the main renal artery of the crossed fused right kidney. The laboratory tests pertinent to a renovascular hypertension and the findings of the captopril radioisotope renography were not conclusive in his case. Although currently the stenotic lesion does not seem to contribute to the elevated blood pressure, it may prove to be so during the followup. So, a close followup was scheduled for our patient.

In crossed fused ectopic kidneys, the vascular anatomy is grossly aberrant and the crossed ectopic kidney generally demonstrates a decreased function. In accordance with the literature, the crossed ectopic right kidney of our patient also had a decreased renal function. In approximately 25% of the CFRE cases, the arteries originate from the superior abdominal aorta, whereas in the remainder the origin is from the inferior abdominal aorta or the iliac arteries [1, 4, 6, 7]. In our case, the origin of the main renal arteries was from the ipsilateral common iliac arteries, respectively.

In the absence of complications and associating additional abnormalities, the prognosis for a patient with CFRE is very good. There are no specific primary treatment approaches for the management of CFRE. Treatment of the associated pathologies is indicated which are most frequently nephrolithiasis and vesicoureteral reflux [6]. It is important to remember that a thorough understanding of the aberrant anatomy is vital before planning of any surgical intervention in the renal region. An angiography is certainly recommended before the surgical intervention. Finally, despite a significantly rare congenital renal abnormality, CFRE should be remembered in cases of renal ectopia with both kidneys located in close proximity.
